# Pilonidal sinus: an overview of historical and current management modalities

**DOI:** 10.1007/s13304-024-01799-2

**Published:** 2024-03-25

**Authors:** Adrian Tam, Christopher J. Steen, Jonathan Chua, Raymond J. Yap

**Affiliations:** 1 Department of General Surgery, Eastern Health, Maroondah Hospital, 1-15 Davey Drive, Ringwood, Victoria 3135 Australia; 2 Department of Surgery, Cabrini Monash University, Melbourne, Victoria Australia; 3 Department of Surgery, Cabrini Monash University, Cabrini Health Australia, Melbourne, Australia

**Keywords:** Pilonidal disease, Pilonidal sinus, Pilonidal abscess, Colorectal surgery

## Abstract

Pilonidal disease is a common condition that commonly affects the younger adult population and is often seen in both the general practice and the hospital setting. Multiple treatment methods have gained and lost popularity over the last several decades, but more recent intervention principles show promising results. This article details the different methods of managing acute and chronic pilonidal disease ranging from treatments in the primary care setting to those in hospital theatres, with special attention to newer modalities of minimally invasive interventions. As a chronic illness that often affects those of working age, pilonidal disease can confer significant morbidity especially, but not limited to, a substantial amount of time off work. Treatment of chronic disease in particular, has evolved from midline techniques to off-midline techniques, with more recent developments offering promising solutions to reduce acute flare ups and hasten recovery time.

## Introduction

In 1833, Herbert Mayo first described the pilonidal sinus as a blind-ending sinus containing hair in the sacrococcygeal region [1]. Almost 200 years later, the pathophysiology of pilonidal disease remains contentious. The works of Karydakis and Bascom have played a significant role in the shift toward viewing pilonidal disease as an acquired disease, rather than a congenital defective remnant of the neural tube [2]. Bascom theorized that the initial insult leading to pilonidal disease is a hair follicle that becomes distended with keratin, triggering inflammation, edema, and infection, after which invasion of hair into the resultant cavity is a secondary event [3]. Karydakis meanwhile proposed three main causative principles: loose hair in the gluteal cleft region that burrows through the skin, becoming deeply embedded due to negative pressure caused by the tightening and lifting of overlying skin from the underlying fascia, and some natural skin vulnerability of the natal cleft itself [3, 4]. The common theme of entrapped hair or debris, either as the primary event or secondary event supported by histologic analysis of excised pilonidal sinus tracts, revealing inflammation that results from keratin plugs and debris, and that the classic midline pits forming due to hair burrowing into the gluteal cleft [5].

Even more contentious are the treatment pathways for chronic pilonidal disease, and despite decades of publications, there remains no single technique that has consistently proven to be superior to others [6, 7].

The aim of this review is to provide a comprehensive overview of the historical and contemporary management strategies for pilonidal sinus disease. This analysis will delineate the various modalities suitable for both community and hospital settings, addressing the distinctive needs of acute and chronic presentations of the condition.

## Acute pilonidal infections

Acutely inflamed pilonidal infections require incision and drainage of all embedded inflammatory debris and hair. Deep wounds in the gluteal cleft have a high chance of poor wound healing due to the friction in the cleft upon movement, as well as constant lateral traction while sitting [3]. Therefore, the convention is to make as lateral a cut as possible when incising and draining an acute infection [8]. In cases where midline pits can be seen, excising and laying pits open at the time of incision and drainage can reduce recurrence. Unfortunately, many midline pits are undetectable until after local edema has settled. Currently recurrence rates for conventional incision and drainage varies from 11 to 67% [9].

While antibiotics may be used as an adjunct in acute infections while awaiting definitive drainage, they should not be used as a standalone therapy in the treatment of acute abscess infections [10, 11].

The predominant microorganisms identified in pilonidal abscesses include mixed anaerobic species (61.6%) followed by skin flora (20%). Notably, 13.4% of cases exhibit no microbial growth [12]. Metronidazole remains the classical choice for anaerobic coverage, while skin flora, predominantly comprised by streptococcus and staphylococcus, can be effectively addressed with a penicillin or a cephalosporin. Co-amoxiclav offers an alternative to cover both entities [13]. Clinicians are advised to prioritize adherence to regional sensitivities in antibiotic selection.

## Chronic pilonidal disease

Research suggests that pilonidal disease may frequently be self-limiting and cases after the age of 40 are rare [14, 15]. Additionally, a 2018 meta-analysis has indicated that only half recur within 5 years [16]. Therefore, incision and drainage alone is a reasonable option for those patients with infrequent acute episodes. Patients who suffer from frequent and severe exacerbations may potentially benefit from more invasive sinus and cavity excision techniques. However, it is important to note that a multitude of medical and surgical therapeutic modalities are currently practiced for the management of chronic disease, underscoring the absence of any single superior therapy.

### Antibiotics

As is the case with acute infections, there is no evidence supporting antibiotic use as a standalone therapy except possibly in the perioperative period and only in immunocompromised patients [17]. Specifically, there is no support for routine peri-operative antibiotics as a prophylaxis in patients with chronic disease. Except in some cases where there is only minor superficial skin infection and in cases of post operative residual cellulitis, where co-amoxiclav is the most commonly prescribed antibiotic [12], the current consensus is that there is no role for either standalone or perioperative use of antibiotics for the average patient [18].

### Community-based treatment of chronic disease

#### Hair removal

As loose hair plays a pivotal role in the development of pilonidal sinus tracts, effective hair removal should help prevent the formation of new tracts and inflammatory exacerbations [19]. However, the actual effectiveness of hair removal remains a subject of debate, with some studies reporting increased recurrence rates in patients performing razor hair removal on themselves [20]. As it is specifically the penetration of loose hairs that is a major factor for disease progression, it can be argued that self-removal of hairs may leave more loose strands behind, potentially increasing disease recurrence. Contrastingly, meticulous depilation and removal of visible loose hairs from the cleft and sinus under vision by clinicians seems to dramatically decrease recurrence rates [21]. This approach, however, is resource-intensive, as depilation must be regular and continued until complete healing is achieved [21]. A systematic review of 35 studies suggested that laser hair removal has some effect in decreasing recurrence rates, although small and heterogenous sample sizes make drawing definitive conclusions difficult [22]. Despite differing opinions on effectiveness, hair removal remains a compelling and easy approach in the management of pilonidal disease.

#### Pit removal

Minimally invasive removal of midline pits with a margin of less than 1 mm, followed by meticulous cleaning and debridement of underlying cavities, is a simple and effective procedure that can be performed either under general anesthesia in theater or local anesthesia in a general practitioner’s office. This procedure be carried out with a scalpel, as first described by Lord and Millar [23], or with a trephine as later popularized by Gips [24]. In Gips’ original paper of 1358 patients, the recurrence rates were 6.5% and 16.2% at 1 and 10 years, respectively [24]. In either method, the wounds are allowed to close by secondary intention. Diligent follow up and good hygiene are essential to prevent further loose hairs from penetrating the open wound [25].

#### Medical obliteration of tracts

The use of phenol as a sclerosing agent to destroy debris within the sinus tract and cavity was first described in 1964 and can be a standalone treatment or a supplementary approach to surgery [26]. Phenol often necessitates multiple treatments and a recent randomized control study (RCT) found no difference in recurrence rates when compared with simple excision alone [27]. Phenol use is restricted in some countries due to its potential to cause local inflammatory responses as well as carry teratogenic risk [11]. Some experts recommend overnight observation because of its potential immediate toxicity.

Fibrin glue was first proposed by Greenberg et al. as a simple adjunct to surgical excision, with its primary function as a space filler rather than sealant, to prevent further accumulation of hair and debris into the wound [28]. A systematic review of 4 RCTs failed to identify its effectiveness as either a monotherapy or complement to surgery [29].

### Hospital-based treatments of chronic disease

#### Midline closures: pit-picking and lateral drainage by Bascom

Initially described as a technique to be performed in an outpatient setting with local anesthetic, the Bascom “pit-picking and lateral drainage” has been used internationally for over 40 years [30]. In modern times, this procedure is more commonly performed under general anesthesia, however the technique itself remains largely unchanged and involves debridement of the underlying cavity to the midline pits via a lateral incision. The midline pits are excised and primarily closed, while the lateral incision is left open for drainage. The 9-year recurrence with this technique is reported to be as high as 29% [31].

#### Midline closures: complete excision of pits and cavities

Complete excision of pits and cavities followed by midline primary closure has been shown in a 2018 meta-analysis to have the highest rates of wound dehiscence, failure, and recurrence [16]. In particular, recurrence rates have been identified to be as high as 67.9% [32]. This is not surprising as the drawbacks of midline closure have long been documented in literature [32, 33]. Consequently, some guidelines now explicitly recommend against primary midline closure [6, 7].

#### Midline closures: healing by secondary intention

Leaving the wound open to heal by secondary intention after an excision is an accepted variation. Patients experience minimal pain, complications are rare, and recurrence rates are generally less than 15% [32]. Obvious disadvantages include extended healing times, which necessitates frequent nursing care and regular outpatient reviews.

#### Off-midline closure and flaps: removal of gluteal cleft where hairs burrow in to form pits

Given the concerns surrounding midline closures, as well as the observation that hairs only insert themselves in the natal cleft [4, 34], Karydakis developed a technique aimed at shifting wounds off the midline while removing the deep gluteal cleft itself.

Karydakis described a method of excision wherein the skin and underlying tissues are fashioned into a flap and advanced across the midline raphe. An elliptical excision is made with the vertices positioned 2 cm lateral to the midline natal cleft, ensuring that the sinus tracts and underlying cavity are included and excised completely [35]. The medial side of the wound is then undermined along the length of wound by a distance of 2 cm and down to fascia, creating a flap that is subsequently lateralized and secured by sutures to the sacral fascia and the lateral wound edge, effectively eliminating any dead space [35]. Karydakis’ original description included a wound drain and keeping the patient in a supine position to discourage hematoma formation in the immediate post operative setting, as well as regular local depilation in the outpatient setting until the wound is healed [35]. This procedure remains the most widely practiced surgical intervention for pilonidal disease in Australia and New Zealand [36].

Roughly 15 years later, Bascom would introduce a modified Karydakis procedure termed the “cleft lift” [37]. Preoperative skin markings are made to assess amount of stretch available in a specific patient’s gluteal region in an attempt to reduce tension on the final suture line [37]. The excision includes the preoperative skin marking resulting in an asymmetric elliptical excision and resultant “lazy S” off-midline suture line. The problematic sinus tracts are excised and the underlying cavity curetted to healthy tissue, leaving a deep layer of fat rather than excising down to sacral fascia, resulting in a shallower and faster-healing wound [37]. Lastly, the mobilized flap is only 2–3 mm deep, allowing a simpler edge to edge skin closure. While comparable recurrence rates have been found between Bascom’s cleft lift and the Karydakis procedure, there appears to be more post-operative wound issues with the former [32]. When examining pooled data from 21 RCTs, Karydakis and Bascom’s procedure had an overall recurrence rate of 2.4% at 24 months and 10.2% at 60 months [32]. However, Bascom’s cleft lift remains less popular, likely due to the challenges with accurate preoperative skin markings [7].

Overall, the technique used for the Karydakis procedure remains variable in multiple aspects. In terms of depth of tissue excision, some surgeons advocate for excision down to fascia as originally described, while some take only 2–3 mm as described by Bascom, with others recommending a depth of roughly 1 cm as described by Kitchen [35], and still others relying on dyes such as methylene blue as a guide. Further variations exist with regard to the use of reinforcing interrupted compression stitches, drain stiches, and drain tubes have all been described.

#### Off-midline plastic techniques: tension free wounds

A variety of rotations flaps described in literature, with rhomboid flaps (such as the Limberg flap) being the most widely recognized, followed by Z-plasty and V–Y plasty [32]. These are similar in principle to the cleft lift, except complex plastic skin rotation flaps rather than simple advancement flaps are used to achieve as close to tension-free suture lines as possible [32]. A recent meta-analysis of 9 RCTs found no difference in recurrence or complication rates between Karydakis’ and Limberg’s respective procedures [32, 38]. However, the study also revealed a high level of variance and heterogeneity of recorded complication rates of 2–33% for wound infection, 1–18% for wound dehiscence, 1–22% for hematoma, and 1–22% for seroma occurrence.

Even more complex perforator flaps have been described in literature [39]. However, the use of these more complex flaps is limited by their technically demands, often requiring longer post-operative hospital stays and patient dissatisfaction due to the disfiguring nature of the resultant scar [39, 40].

Overall, current literature has not conclusively established a single superior excision and off-midline closure technique. The injection of methylene blue into sinus tract to reveal their extent and depth for guiding complete excision has been shown to decrease recurrence rates. A study involving over 200 patients followed up over 15 years found a decreased recurrence rate from 30 to 16% with the use of methylene blue [41].

### Newer techniques

#### Negative pressure dressings

Negative pressure dressings have the ability to bring wound margins closer together, increase blood flow, promote angiogenesis, and enhance granulation, making them a valuable option in pilonidal disease where wound healing is often a significant challenge. Retrospective studies suggest low recurrence and complication rates [42], but comprehensive research on the efficacy of negative pressure dressings in pilonidal disease remains sparse [32].

A small unblinded RCT comparing negative pressure dressings with traditional packing of pilonidal excisions, found significantly faster early healing with negative pressure therapy but no significant difference in the overall time to full healing or time to resume daily activities [43]. Other retrospective studies have also reported non-statistically significant improvements with negative pressure therapy [44]. The largest prospective study, involving 65 patients, showed a statistically significant decrease in post-operative wound complications and recurrence in patients treated with negative pressure therapy [45]. Nevertheless, all current literature on the application of negative pressure is constrained by small, heterogenous sample sizes and short term followed up.

#### Laser ablation of tracts

Sinus laser closure (SiLaC) is a technique described by Dessily in 2017 as a novel application of laser therapy already used for varicose veins [37]. In this day-case procedure, debris and hair are mechanically removed from within the sinus and cavity, followed by the insertion of a radial diode laser probe along the entire length of the cavity to induce destruction of the squamous epithelium to obliterate the tract.

In their initial paper, Dressily et al. demonstrated good success rates, with only one recurrence [46]. Their second paper of 200 patients showed a very quick median healing time of 19.5 days, with recurrence and complication rates (14.9% and 15%, respectively) comparable to other surgical techniques [47]. A systematic review of SiLaC suggested an overall recurrence rate of only 4.7% and complication rate of 10% [47]. Other significant advantages of this technique include reduced intensity and duration of pain, technical ease of application, and shorter hospital stays [48]. A recent study comparing SiLaC with the Limberg flap found similar healing rate and replicated the advantages of shorter operative time, reduced hospital stay, and decreased post operative pain seen in other studies [49].

It is important to note that SiLaC is a blind procedure, and as such, side branches or deep cavities of a sinus can be missed during attempts to obliterate tracts, potentially leaving patients vulnerable to long-term recurrence. Moreover, there is a theoretical possibility that hair and debris deeper in the tracts and cavities may remain in situ.

#### Endoscopic interventions: aimed to clear and obliterate tracts under direct vision

Meinero and Milone addressed this specific shortcoming of SiLaC when they concurrently described an endoscopic approach that allows for the visualization of hair, debris, deeper cavities, and lateral sinus’ that may otherwise be missed [50, 51]. An endoscopic fistuloscope is inserted into the sinus allowing mechanical removal of hair and debris under direct vision, followed by radiofrequency ablation of the tract itself. Necrotic material can further be removed under direct vision with an endobrush or curette.

A multicenter prospective study of 250 consecutive patients undergoing endoscopic intervention produced an early complication rate of zero, mean healing time of 26.7 days, and a mean return to work of only 2 days [52]. They observed a recurrence rate of 5% at 12 months regardless of whether endoscopy was used as the first line treatment, or used after failure of another intervention [52].

Milone subsequently compared their endoscopic technique with the Bascom cleft lift in a randomized control trial of 145 patients. They found complication rates were not statistically significant between the two treatments. Recurrence was also not statistically different (3.9% versus 5.8% respectively). However, mean return to work was substantially shortened to 1.6 from 8.2 days in the endoscopic group compared to the traditional off-midline group [53].

Endoscopic intervention may even be effective in the acute abscess phase of pilonidal disease [54, 55]. A small 1 cm incision is made at the area of maximal fluctuance and pus drained, followed by fistuloscopy through the same incision to washout, debride, and fulgurate the abscess cavity and deeper tracts under direct vision [54, 55]. When compared to traditional incision and drainage, endoscopic intervention significantly reduced the median duration of complete wound healing from 28 to 16 days. Return to work was also reduced from 4 to 2.5 weeks [54]. One study reported a reduction of 6-month recurrence from 6 to 0 when comparing conventional incision to endoscopic drainage [54]. Although both papers suffered from very small sample sizes, their overall results of comparable complication rates, similar or improved recurrence rates, and shortened healing times have been reported by other studies [56–58].

Further variations to endoscopic therapy have been described, including use of bipolar resectoscope [59], removal of hair under direct vision while flushing with an angio-catheter [60], and by adding laser therapy to the original endoscopic approach described above [61]. Early data on the latter demonstrated faster healing, sooner return to work, and better patient comfort with laser assisted endoscopic therapy [61]. However, the data comes from a retrospective study with a short follow up time of only 9 months, small sample size of 24 patients, and was published by the same authors who first described the technique. None of these new variations on the endoscopic approach have been independently reviewed by independent researchers.

A potential drawback is that these minimally invasive treatments do not address Karydakis’ third factor of pilonidal disease, namely the underlying skin vulnerability of the natal cleft. Theoretically, further loose hairs may form new midline pits and sinuses, regardless of the effectiveness of laser or endoscopic intervention on current pits and cavities. Long term data are needed to answer this question.

## Discussion

The debate between acquired and congenital theories for pilonidal disease has yet to be settled, although consensus now leans mainly toward its classification as an acquired disease. The exact pathophysiology remains even more a matter of controversy. Although the common core principle of chronic retention of keratin or debris exists, ongoing debate remains between Bascom’s hypothesis of retained hair as a secondary insult and Karydakis’ theory that burrowing of loose hair is the primary insult, which includes, Stelzner’s theory of dermatopathy [62].

Although it is difficult for current literature to definitively establish a gold standard intervention for pilonidal disease, we now have enough data to recommend against certain interventions. Namely, midline closures should be avoided, and this recommendation is endorsed in several countries. Similarly, antibiotics play a very limited role in the treatment and prevention of pilonidal disease, and should rarely be used as a standalone therapy. Furthermore, the obliteration of tracts with phenol and, especially, fibrin as standalone treatments, appear to be less effective than other surgical treatments.

Minimally disfiguring and technically simpler procedures, such as Gip’s procedure, are reasonable first-line treatments in chronic pilonidal disease. While recurrence rates may appear to be higher compared to off-midline flap techniques, these procedures offer faster healing times, allowing earlier return to work, and provide better cosmesis by preserving the natural contour of the gluteal cleft is left intact. Off-midline flaps may be better left for complex disease or after failure of other surgical management. However, current literature has yet to definitively establish a superior off-midline closure technique. Moreover, the closure techniques themselves are technically diverse and nuanced, so surgeon familiarity with each procedure undeniably plays a significant role in success and recurrence.

In an effort to reduce healing times, minimally invasive techniques, including laser and endoscopic approaches, have now been introduced. In particular, endoscopy seems to be viable for both acute and chronic phases of pilonidal disease. In today’s climate of minimally invasive surgeries, these techniques provide an attractive step-up approach before committing to more expansive surgical excisions. These techniques have comparable short-term recurrence and complications rates, but more research to accurately assess long term recurrence rates is required before wide-spread adoption of this resource intensive approach is likely. The current body of research into endoscopic techniques is lacking in terms of available adequately-powered, quality studies. This is highlighted best in a recent systematic review where Milone et al. found only one randomized control trial out of 38 studies on endoscopic techniques [63]. The review found marked heterogeneity in even the terminology used to describe similar procedures, making the consolidation and analysis of research findings unnecessarily arduous and unreliable.

It is important to note that overall research into treatment of pilonidal disease, in particular chronic disease, remains relatively poor and heterogenous, with small sample size and short follow up. In particular, the variation in follow-up times among studies renders interpretation of long-term success rates between techniques challenging. The lack of RCTs is not limited to studies on endoscopic therapies. A meta-analysis of 93 papers comparing flap techniques found only 9 RCTs, which were all deemed to be of low quality [38]. This may account for the wide range of recurrence rates and complication rates reported in literature [10, 38]. There exists a need for high quality, high-powered, studies with consistently measured outcomes.

Clinicians should not discount simple conservative approaches such as hair removal and good hygiene. This is especially true when presented with a young patient who may not be able afford prolonged periods off work.

The hallmarks of a gold standard pilonidal treatment should encompass effective removal of acute infection, maintenance of low recurrence and complication rates, and swift healing to facilitate earlier return to work. Despite several decades of research and development of novel approaches, no single technique has conclusively met all these criteria. Thus, the choice of intervention remains a matter of individual surgeon familiarity, and tailored to patient characteristics.

## Data Availability

Not applicable.
